# Adverse effects of low serum lipoprotein cholesterol on the immune microenvironment in gastric cancer: a case‒control study

**DOI:** 10.1186/s12944-022-01766-z

**Published:** 2022-12-31

**Authors:** Yi Zou, Xiaoyan Yu, Chenqi Zhou, Chunpeng Zhu, Ying Yuan

**Affiliations:** 1 Department of Pathology, Second Affiliated Hospital Zhejiang University School of Medicine, 310058 Hangzhou, Zhejiang China; 2 Department of Gastroenterology, Second Affiliated Hospital Zhejiang University School of Medicine, 310058 Hangzhou, Zhejiang China; 3 Department of Medical Oncology, Cancer Center, Second Affiliated Hospital Zhejiang University School of Medicine, Zhejiang University, 310058 Hangzhou, Zhejiang China

**Keywords:** Gastric cancer, Lipoprotein cholesterol, Tumor immune microenvironment, Immunometabolism

## Abstract

**Background:**

Cholesterol is crucial for tumor immune microenvironment (TIME) remodeling. Serum lipoprotein cholesterol is closely associated with gastric cancer (GC) progression, but whether it affects TIME remodeling is unknown.

**Methods:**

GC patients with differential serum high-density lipoprotein (HDL) or low-density lipoprotein (LDL) cholesterol levels were collected. After balancing the baseline, immunohistochemical staining was performed on serial whole-tissue sections to detect B-cell and T-cell subsets, macrophages, and PD-L1. Features of tertiary lymphoid structures (TLSs) and the extra-TLS zone, including TLS distribution and maturation, immune cell density, and PD-L1 expression, were measured by annotating TLSs or regions of interest (ROIs) in the extra-TLS zone.

**Results:**

A total of 9,192 TLSs and over 300 ROIs from 61 patients were measured. Compared to HDL-normal patients, HDL-low patients had a decreased secondary-TLS fraction or density but an elevated NK-cell density in the extra-TLS zone. Compared to LDL-normal patients, LDL-low patients had a higher ratio of PD-1 + T follicular helper cells to CD20 + B cells in TLSs, a higher ratio of PD-1 + T cells to CD8 + T cells and increased PD-1 + T-cell density in the extra-TLS zone. Different correlations were found in groups with differential HDL or LDL levels. Cell dynamics in the immune response were weaker in patients with low lipoprotein cholesterol. TLS parameters reached their peak earlier than those of the extra-TLS zone along with tumor progression.

**Conclusion:**

Low serum lipoprotein cholesterol caused adverse effects on antitumor immunity in GC. Lipid management or immunometabolic drugs deserve more attention.

**Supplementary Information:**

The online version contains supplementary material available at 10.1186/s12944-022-01766-z.

## Introduction

Gastric cancer (GC) is the second most common malignancy of the digestive system worldwide [1] and the leading cause of cancer deaths in the digestive tract in China [2]. With the availability of novel therapeutics, such as immunotherapy, the outcomes of cancer patients have been improved significantly in recent years. However, compared to other cancer types, the outcome of advanced GC patients is still unsatisfactory.

Dietary intake, especially lipids, and serum lipid levels are proven to be associated with abundant cardiovascular, metabolic, and autoimmune diseases, such as coronary heart disease, hypertension, diabetes, and rheumatoid arthritis [3–7]. Currently, increasing evidence has shown that serum lipids are also correlated with the prevalence and behaviors of various tumors, especially those of the digestive system, including GC, pancreatic cancer, and colorectal cancer [8–11]. However, to date, most related studies have focused on the direct effect of lipids on tumor cells, but the relationship between serum lipids and nontumorous components in the tumor microenvironment, especially immune cells and structures, is ill defined.

As an important part of the TIME, tumor-infiltrating lymphocytes are related to tumor progression and patient survival [12, 13]; however, as local schools of lymphocytes, tertiary lymphoid structures (TLSs) have shown more profound significance in antitumor activities. TLSs are organized immune structures, presenting as aggregates of immune cells [14], which provide the main site for B-cell maturation for humoral immunity and are also involved in T-cell activation [15]. The features of TLSs, including their formation, spatial organization, maturation, and cellular composition, were found to be correlated with tumor invasion, node metastasis, antitumor therapeutic effect, and clinical outcome in GC and other tumors [16–18].

Immunometabolism is one of the pivotal factors in TIME modulation and remodeling. The different components and concentrations of nutrients or metabolites affect immune cell function and help determine the outcome of the battle between tumors and host immunity [19]. Lipids, such as cholesterol, play essential roles in T- and B-cell immunity; for example, the amount of membrane cholesterol impacts T-cell activation and function [20], and the downstream products of cholesterol are necessary for B-cell migration [21]. However, due to the activity of tumor cells, a disadvantageous environment with hypoxia, lactate accumulation, and oxidative stress impaired the *de novo* lipid synthesis of immune cells [22, 23], which makes them more sensitive to the lipid dynamics in the tumor microenvironment. It has been well demonstrated that dyslipidemia, especially abnormal levels of serum lipoprotein cholesterol, is closely associated with GC occurrence and progression [24–26]. However, the relationship between serum lipoprotein cholesterol and TIME was unclarified in GC.

Hence, in the present study, based on whole-tissue sections, the TIME features were compared between baseline-controlled GC cohorts who had differential preoperative high-density lipoprotein cholesterol (HDL-C) or low-density lipoprotein cholesterol (LDL-C) levels. Comprehensively, we studied both main immune compartments, the TLS zone, which is just the TLSs, and the extra-TLS zone, with a focus on TLS distribution and maturation, features of intra-TLS immune cells (tICs) and extra-TLS immune cells (eICs), immune ratios, and tumor PD-L1 expression, seeking to reveal the relationship between serum lipoprotein cholesterol and the TIME of GC.

## Materials and methods

### Patient collection and propensity-score matching

GC patients’ medical records collected by our team from the Second Affiliated Hospital Zhejiang University School of Medicine between 2016 and 2019 were reviewed, and those who met all the following criteria were enrolled: (i) a radical gastrectomy was performed; (ii) whole-tissue blocks of the tumor were available; (iii) neoadjuvant chemotherapy, radiotherapy, and/or immunotherapy were not applied; and (iv) preoperative serum lipid levels had been determined.

According to the data on preoperative HDL-C or LDL-C levels, patients were divided into HDL-low (< 1.15 mmol/L) and HDL-normal (≥ 1.15 mmol/L and < 1.68 mmol/L) groups (due to the low proportion of GC with elevated HDL-C [27], those patients were not included in this study) or LDL-low (< 2.10 mmol/L), LDL-normal (≥ 2.10 mmol/L and < 3.10 mmol/L) and LDL-high (≥ 3.10 mmol/L) groups.

To balance the baseline, first, patients’ demographic features, including sex and age, and clinicopathologic features, including tumor location, tumor size, histological differentiation, tumor invasion (T), node metastasis (N), distal metastasis (M), and pathological stage (pStage), were compared between groups with differential HDL-C or LDL-C levels. Then, propensity-score matching (PSM) was performed when any significant difference was found in the comparisons above based on variables with significant differences.

The study was conducted in accordance with the Declaration of Helsinki (as revised in 2013) and approved by the Review Board of Second Affiliated Hospital of Zhejiang University School of Medicine (2020-ERR-031); patient consent was waived by the institutional review boards, as this study was retrospective, and patient information was protected by a blind method.

## Immunohistochemical staining

For each case, one formalin-fixed and paraffin-embedded tissue block was selected by reviewing the H&E slides for IHC staining. This block should contain the whole section of a tumor, including the tumor, invasive margin, and adjacent normal tissues.

IHC staining for 11 markers, including CD20 (OTI4B4, Ready-to-use, ZSGB-Bio), IgD (EPR6146, 1:3500, Abcam), CD21 (EP3093, 1:500, Abcam), CD23 (EPR3617, 1:400, Abcam), CD8 (SP16, Ready-to-use, ZSGB-Bio), FOXP3 (EPR22102-37, 1:250, Abcam), PD-1 (EPR4877 [2], 1:500, Abcam), EOMES (EPR21950-241, 1:1000, Abcam), NCR1 (EPR22403-57, 1:1000, Abcam), CD163 (EPR19518, 1:500, Abcam) and PD-L1 (E1L3N, 1:200, Cell Signaling Technology), was performed on the serial whole-tissue sections of each case by a two-step polymer-based detection system (PV-8000, ZSGB-Bio). After quality control, IHC and H&E slides were scanned by Aperio Digital Pathology Slide Scanners (Aperio Technologies, Vista, CA, USA) with a magnification of 200X for subsequent analysis.

## Quantitative assessment of TLSs

TLSs were defined as CD20 + cell aggregates larger than 10,000 µm^2^, of which location, count, and area were evaluated based on CD20 staining with QuPath v0.3.0 software (University of Edinburgh, UK) by one-by-one annotation. The TLS area to tumor area ratio (TLS/tumor) and the single TLS size (the mean area of all TLSs in a tumor) were also calculated. According to CD21 and CD23 staining, TLSs in a tumor were classified into three developmental stages: early (CD21-CD23-), primary (CD21 + CD23-), and secondary (CD21 + CD23+) stages [28]. The fraction and density of TLSs at each stage were calculated as the count of TLSs at a specific stage divided by the total TLS count or tumor area, respectively. Tumor areas were annotated and measured on H&E slides.

Intra-TLS immune cells (tICs) studied here included CD20 + pan B cells (tB cell-pan), IgD + naïve B cells (tB cell-naïve), CD8 + cytotoxic T lymphocytes (tCTLs), FOXP3 + T follicular regulatory (tTfr) cells, PD-1 + T follicular helper (tTfh) cells, and EOMES + exhausted T (tTeom) cells. The total tIC count, positive tIC count, tIC fraction (the count ratio of a certain kind of tIC to all tICs), and tIC density per mm^2^ in TLSs were quantified with QuPath v0.3.0 software (University of Edinburgh, UK). The tIC density per mm^2^ in a tumor was calculated as the positive tIC count divided by the tumor area. The immune ratios, including tB cell-naïve/tB cell-pan, tTfh/tB cell-naïve, tTfh/tB cell-pan, tTfr/tB cell-naïve, tTfr/tB cell-pan, and tTfr/tTfh, were calculated using the corresponding cell counts.

## Quantitative assessment of the extra-TLS zone

According to the infiltration patterns of CD8 + T cells, the immune environment of the extra-TLS zone was classified into immune-excluded, inflamed, or immune desert phenotypes [29]. The immune-excluded phenotype referred to tumors with CD8 + T cells restrained in the stroma, without any infiltrations into tumor nests; the inflamed or immune desert phenotype referred to tumors with a density of tumor-infiltrating CD8 + T cells higher than the upper quartile or lower than the lower quartile.

Under a magnification of 100X, five tumor regions without TLSs were selected randomly as representatives of the extra-TLS zone. The extra-TLS immune cells (eICs) studied here included CD20 + pan B cells (eB cell-pan), IgD + naïve B cells (eB cell-naïve), CD8 + cytotoxic T lymphocytes (eCTLs), FOXP3 + regulatory T (eTreg) cells, PD-1 + exhausted T (eTpd-1) cells, EOMES + exhausted T (eTeom) cells, NCR1 + natural killer (eNK) cells, and CD163 + tumor-associated macrophages (eTAMs). Positive eIC count and eIC density in tumors were quantified with QuPath v0.3.0 software (University of Edinburgh, UK). The immune ratios, including eB cell-naïve/eB cell-pan, eTreg/eCTL, eTpd-1/eCTL, and eTeom/eCTL, were calculated using the corresponding cell counts.

The expression of PD-L1 was determined using combined positive scores (CPS) and PD-L1 + cell density [30]. Briefly, CPS was the number of PD-L1-stained cells (tumor cells, lymphocytes, macrophages) divided by the tumor cell count. PD-L1 + cell density was the number of PD-L1-stained cells divided by tumor area.

## Statistical analyses

The distributions of categorical variables, such as demographics, clinicopathological features, TLS presence and location, and tumor immune phenotypes, were compared using the 𝜒2 test or Fisher’s exact test. Quantitative variables following abnormal distributions were presented as medians (range) and compared with the U test (when two groups were compared) or Kruskal‒Wallis test (when more than two groups were compared). PSM was performed between groups based on the differential variable(s) by the matching ratio of 1:1 using the algorithm of nearest neighbors with calipers of width equal to 0.2 in R software v4.1.2 (The R Project for Statistical Computing, Vienna, Austria). Correlation analysis was performed with the Spearman test. A *P* value less than 0.1 was considered statistically significant in baseline comparisons; for other tests, a *P* value less than 0.05 was considered statistically significant. Statistical analyses were performed with GraphPad Prism 9 (GraphPad Software LLC., San Diego, CA, USA) and SPSS 26.0 (SPSS Inc., Chicago, IL, USA).

## Results

### Patient baseline

A total of 61 eligible patients with available whole-tissue blocks were enrolled in this study. To ultimately balance the patient baseline, PSM was performed when the *P* value was < 0.1 in baseline comparisons. Before PSM, significant differences were found between groups divided by HDL-C levels in sex and node metastasis distributions. After PSM, 23 pairs of matched patients were obtained, and the variables, including but not limited to sex and node metastasis, were significantly more balanced. Of the matched patients, 78.3% were male, and 69.6% were over 60 years old. More than half of the patients had tumors in the middle and lower 1/3 of the stomach, 65.2% of tumors were larger than 3 cm, and moderate/poor differentiation was the main histological type. Most tumors were graded as T1-T3, and 56.5% had node metastasis, but distal metastasis was not observed. All matched patients were at pStage I-III with a relatively even distribution (Table 1).

**Table 1:** Baseline of patients with differential HDL-C levels

Factor	Before matching			After matching		
	HDL-low *n* = 31	HDL-normal *n* = 30	*P*	HDL-low *n* = 23	HDL-normal *n* = 23	*P*
**Sex**						
Male	25 (80.6)	18 (60.0)	0.097	18 (78.3)	18 (78.3)	1.000
Female	6 (19.4)	12 (40.0)		5 (21.7)	5 (21.7)	
Age						
< 60 yr	8 (25.8)	12 (40.0)	0.283	7 (30.4)	7 (30.4)	1.000
≥ 60 yr	23 (74.2)	18 (60.0)		16 (69.6)	16 (69.6)	
Tumor location						
Upper 1/3	6 (19.4)	3 (10.0)	0.223	5 (21.7)	3 (13.0)	0.536
Middle 1/3	8 (25.8)	14 (46.7)		6 (26.1)	9 (39.1)	
Lower 1/3	17 (54.8)	13 (43.3)		12 (52.2)	11 (47.8)	
Tumor size, cm						
< 3 cm	8 (25.8)	13 (43.3)	0.184	8 (34.8)	8 (34.8)	1.000
≥ 3 cm	23 (74.2)	17 (56.7)		15 (65.2)	15 (65.2)	
Histological differentiation						
Well	3 (9.7)	1 (3.3)	0.779	2 (8.7)	1 (4.3)	0.818
Moderate	13 (41.9)	13 (43.3)		9 (39.1)	11 (47.8)	
Poor	15 (48.4)	16 (53.3)		12 (52.2)	11 (47.8)	
T						
T1	6 (19.4)	7 (23.3)	0.849	6 (26.1)	6 (26.1)	1.000
T2	9 (29.0)	7 (23.3)		5 (21.7)	5 (21.7)	
T3	12 (38.7)	10 (33.3)		9 (39.1)	9 (39.1)	
T4	4 (12.9)	6 (20.0)		3 (13.0)	3 (13.0)	
**N**						
Negative	10 (32.3)	17 (56.7)	0.073	10 (43.5)	10 (43.5)	1.000
Positive	21 (67.7)	13 (43.3)		13 (56.5)	13 (56.5)	
M						
Negative	31 (100.0)	30 (100.0)	-	23 (100.0)	23 (100.0)	-
Positive	0	0		0	0	
pStage						
I	9 (29.0)	10 (33.3)	0.276	9 (39.1)	7 (30.4)	0.607
II	10 (32.3)	14 (46.7)		6 (26.1)	10 (43.5)	
III	12 (38.7)	6 (20.0)		8 (34.8)	6 (26.1)	

Bold: variables for propensity-score matching

Between groups divided by LDL-C levels, there was no significant difference in demographic and clinicopathological features, and all 61 patients were included in the following analysis. Of these patients, 54.5-76.2% were male, and more than 60.0% were over 60 years old. A total of 81.0-90.9% of the tumors were located in the middle and lower 1/3 of the stomach, 55.2-76.2% were larger than 3 cm, and moderate/poor differentiation was the main histological type. A total of 72.7-95.2% of the tumors were graded as T1-T3, and 51.7-63.6% had node metastasis, but distal metastasis was not observed. All patients were distributed at pStage I-III without significant differences among groups (Table 2).

**Table 2:** Baseline of patients with differential LDL-C levels

Factor	LDL-low *n* = 21	LDL-normal *n* = 29	LDL-high *n* = 11	*P*^†^
Sex				
Male	16 (76.2)	21 (72.4)	6 (54.5)	1.000/0.451
Female	5 (23.8)	8 (27.6)	5 (45.5)	
Age				
< 60 yr	5 (23.8)	11 (37.9)	4 (36.4)	0.365/1.000
≥ 60 yr	16 (76.2)	18 (62.1)	7 (63.6)	
Tumor location				
Upper 1/3	4 (19.0)	4 (13.8)	1 (9.1)	0.632/0.702
Middle 1/3	5 (23.8)	11 (37.9)	6 (54.5)	
Lower 1/3	12 (57.1)	14 (48.3)	4 (36.4)	
Tumor size, cm				
< 3 cm	5 (23.8)	13 (44.8)	3 (27.3)	0.149/0.473
≥ 3 cm	16 (76.2)	16 (55.2)	8 (72.7)	
Histologic differentiation				
Well	2 (9.5)	2 (6.9)	0	1.000/0.855
Moderate	9 (42.9)	13 (44.8)	4 (36.4)	
Poor	10 (47.6)	14 (48.3)	7 (63.6)	
T				
T1	3 (14.3)	8 (27.6)	2 (18.2)	0.205/0.622
T2	8 (38.1)	7 (24.1)	1 (9.1)	
T3	9 (42.9)	8 (27.6)	5 (45.5)	
T4	1 (4.8)	6 (20.7)	3 (27.3)	
N				
Negative	9 (42.9)	14 (48.3)	4 (36.4)	0.778/0.723
Positive	12 (57.1)	15 (51.7)	7 (63.6)	
M				
Negative	21 (100.0)	29 (100.0)	11 (100.0)	-
Positive	0	0	0	
pStage				
I	6 (28.6)	11 (37.9)	2 (18.2)	0.770/0.459
II	10 (47.6)	10 (34.5)	4 (36.4)	
III	5 (23.8)	8 (27.6)	5 (45.5)	

† LDL-low vs. LDL-normal / LDL-high vs. LDL-normal

## TLS distribution

**Fig. 1:**
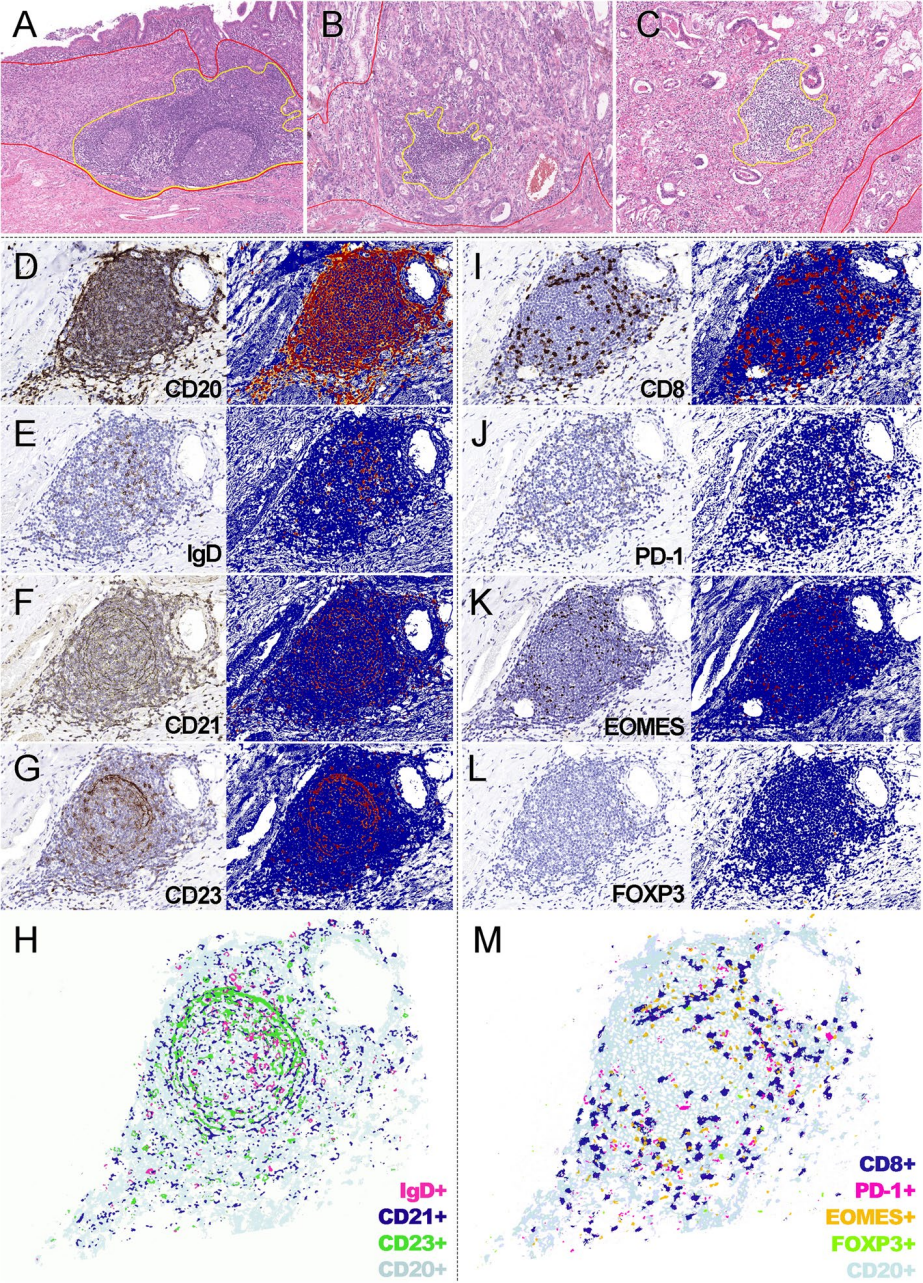
Morphology of TLSs in GC (**A**) A representative peritumoral TLS (H&E, 200×); **B** A representative intratumoral TLS (H&E, 200×); **C** A representative stromal TLS (H&E, 200×); **D** The distribution of pan B cells in a TLS (CD20, 200×); (**E**) The distribution of naïve B cells in a TLS (, 200×); **F** The distribution of follicular dendritic cells in a TLS (CD21, 200×); **G** The distribution of follicular dendritic cells in a TLS (CD23, 200×); **H** Merged image of pan and naïve B cells and follicular dendritic cells (200×); **I** The distribution of CTLs in a TLS (CD8, 200×); **J** The distribution of tTfh cells in a TLS (PD-1, 200×); **K**. The distribution of exhausted T cells in a TLS (EOMES+, 200×); **L**. The distribution of tTfr cells in a TLS (FOXP3, 200×); **M**. merged image of T cells (200×)

TLSs were frequently observed in our cohort, presenting as lymphocyte aggregates on H&E slides (Fig. 1 A-C) or nests composed predominantly of B cells on IHC slides (Fig. 1D-M).

In groups with differential HDL-C levels, TLSs were present in 91.3% of the tumors, with a slightly lower proportion of TLS + tumors in the HDL-low group. Most TLSs were situated at the stromal or peritumoral location without a significant difference between the HDL-low and HDL-normal groups (Supplementary Table 1).

In groups with differential LDL-C levels, 93.4% of the tumors harbored TLSs, with a slightly lower proportion of TLS + tumors in the LDL-low group. The tumor stroma and peritumoral tissue were also the most common sites for TLSs. There was no significant difference in TLS presence or location between the LDL-normal and LDL-low or LDL-high groups (Supplementary Table 1).

## Delayed TLS maturation and a higher eNK-cell density in patients with low HDL levels

**Fig. 2:**
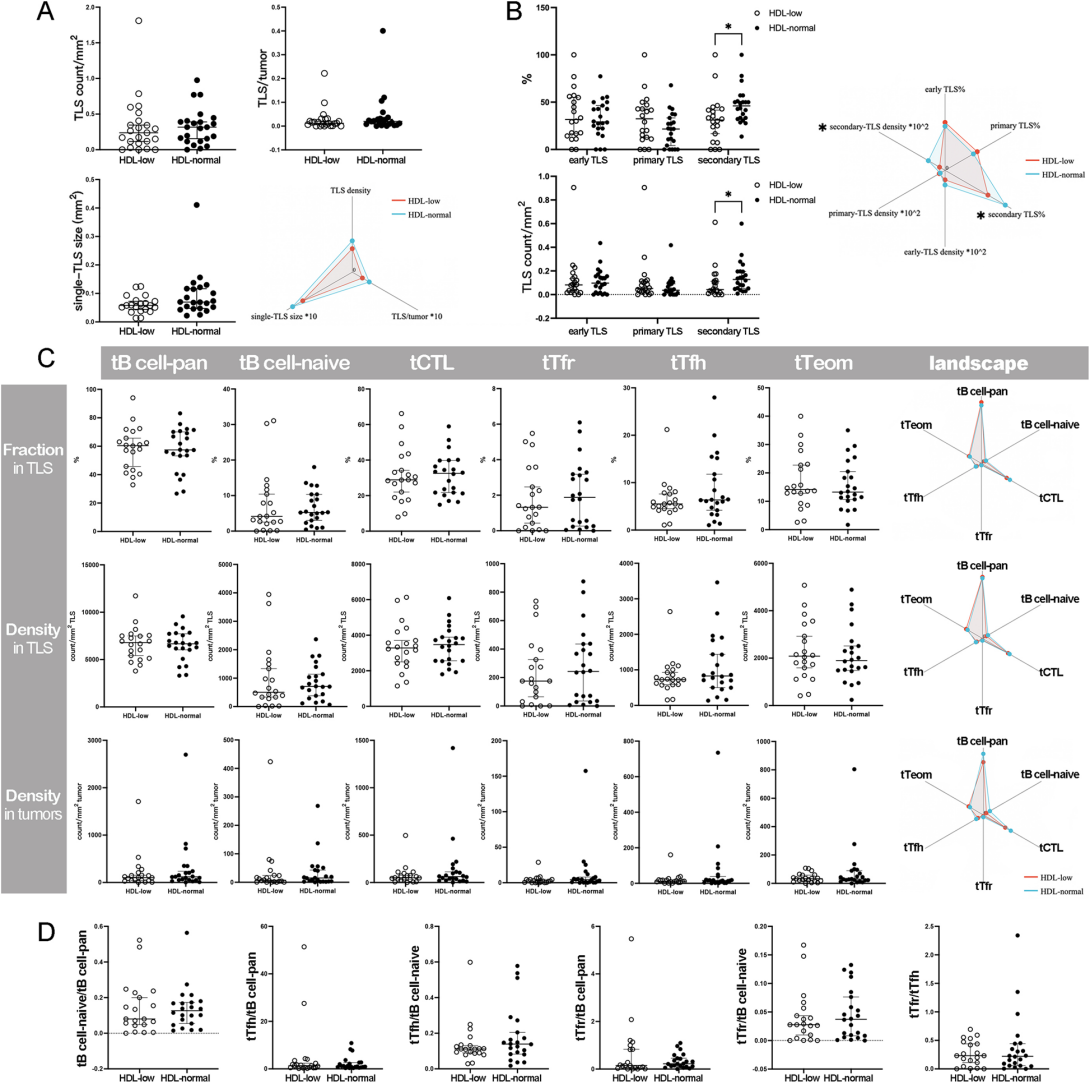
Comparison of TLSs between groups with differential HDL-C levels (**A**) Comparisons of TLS basic features; **B** Comparison of TLS maturation; **C** Comparison of tICs; **D** Comparison of immune ratios. *P < 0.05

A total of 6352 TLSs were annotated and measured in the HDL-low and HDL-normal groups. The median TLS count per tumor was 13.5 (range: 1.0–56.0) in the HDL-low group and 17.5 (range: 2.0–66.0) in the HDL-normal group. The median TLS density was 0.28/mm^2^ (range: 0.01/mm^2^-1.81/mm^2^) in the HDL-low group and 0.32/mm^2^ (range: 0.02/mm^2^-0.98/mm^2^) in the HDL-normal group. However, no significant difference was found either in the features above or in TLS/tumor and single-TLS size (Fig. 2 A). However, TLSs in the HDL-low group showed a prominently lower degree of maturation: the fraction and density of secondary TLSs were significantly lower in the HDL-low group (Fig. 2B).

In the comparisons of tICs, the fractions and densities of tB cell-pan, tB cell-naïve, tCTLs, tTfr cells, tTfh cells, and tTeom cells were comparable between groups (Fig. 2 C). Similarly, there was no significant difference in the ratios of tB cell-naïve/tB cell-pan, tTfh/tB cell-naïve, tTfh/tB cell-pan, tTfr/tB cell-naïve, tTfr/tB cell-pan, and tTfr/tTfh (Fig. 2D).

**Fig. 3:**
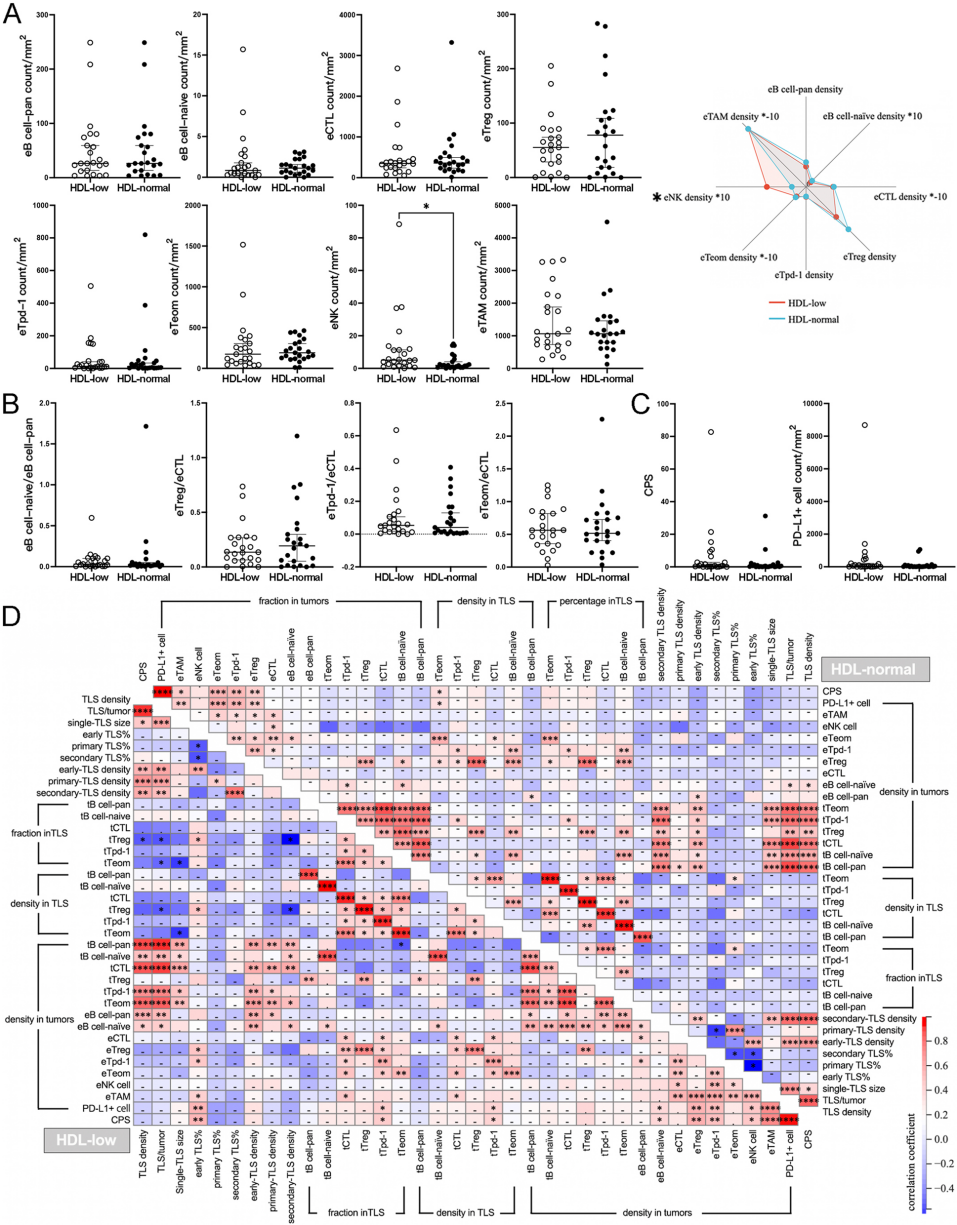
Comparison of the extra-TLS zone between groups with differential HDL-C levels and correlation analysis. (**A**) Comparison of eICs; **B** Comparison of immune ratios; **C** Comparison of PD-L1 expression; **D** Correlations between TIME parameters in the HDL-low or HDL-normal group. *P < 0.05

In the extra-TLS zone, unexpectedly, eNK-cell density significantly increased in tumors of the HDL-low group, while the densities of other eICs, such as eB cell-pan, eB cell-naïve, eCTLs, eTreg cells, eTpd-1 cells, eTeom cells, and eTAM, were comparable between groups (Fig. 3, A). The ratios of eB cell-naïve/eB cell-pan, eTreg/eCTL, eTpd-1/eCTL, and eTeom/eCTL in the HDL-low group were also similar to those in the HDL-normal group (Fig. 3B). In addition, there was no significant difference in PD-L1 expression (Fig. 3 C) or the distribution of immune phenotypes between groups (Supplementary Table 2).

In either the HDL-normal or HDL-low group, abundant correlations were found within an immune compartment or between the two compartments. However, in each group, some exclusive correlations were also seen, particularly regarding the significantly differential variables in the comparisons above. For example, in the HDL-low group, secondary TLS density was negatively correlated with the fraction or density of tTfr cells, and the density of eNK cells was positively correlated with the density of eTpd-1 cells, eTeom cells, eTAMs or PD-L1 + cells and CPS, while these correlations were absent in the HDL-normal group. Instead, in the HDL-normal group, secondary TLS density showed a strong positive correlation with tTfh cell density (Fig. 3D).

Higher ratios of tTfh/tB cell-pan and more exhausted extra-TLS T cells in patients with low LDL levels.

In the LDL-low, LDL-normal and LDL-high groups, 9192 TLSs were annotated and measured. The median count of TLSs per tumor was 16.0 (range: 4.0–52.0), 14.0 (range: 1.0–66.0), and 20.0 (range: 2.0–51.0) in the LDL-low, LDL-normal, and LDL-high groups, respectively. The median TLS density was 0.26/mm^2^ (range: 0.06/mm^2^-1.81/mm^2^), 0.31/mm^2^ (range: 0.01/mm^2^-0.98/mm^2^), and 0.20/mm^2^ (range: 0.02/mm^2^-0.61/mm^2^) in the LDL-low, LDL-normal, and LDL-high groups, respectively.

**Fig. 4:**
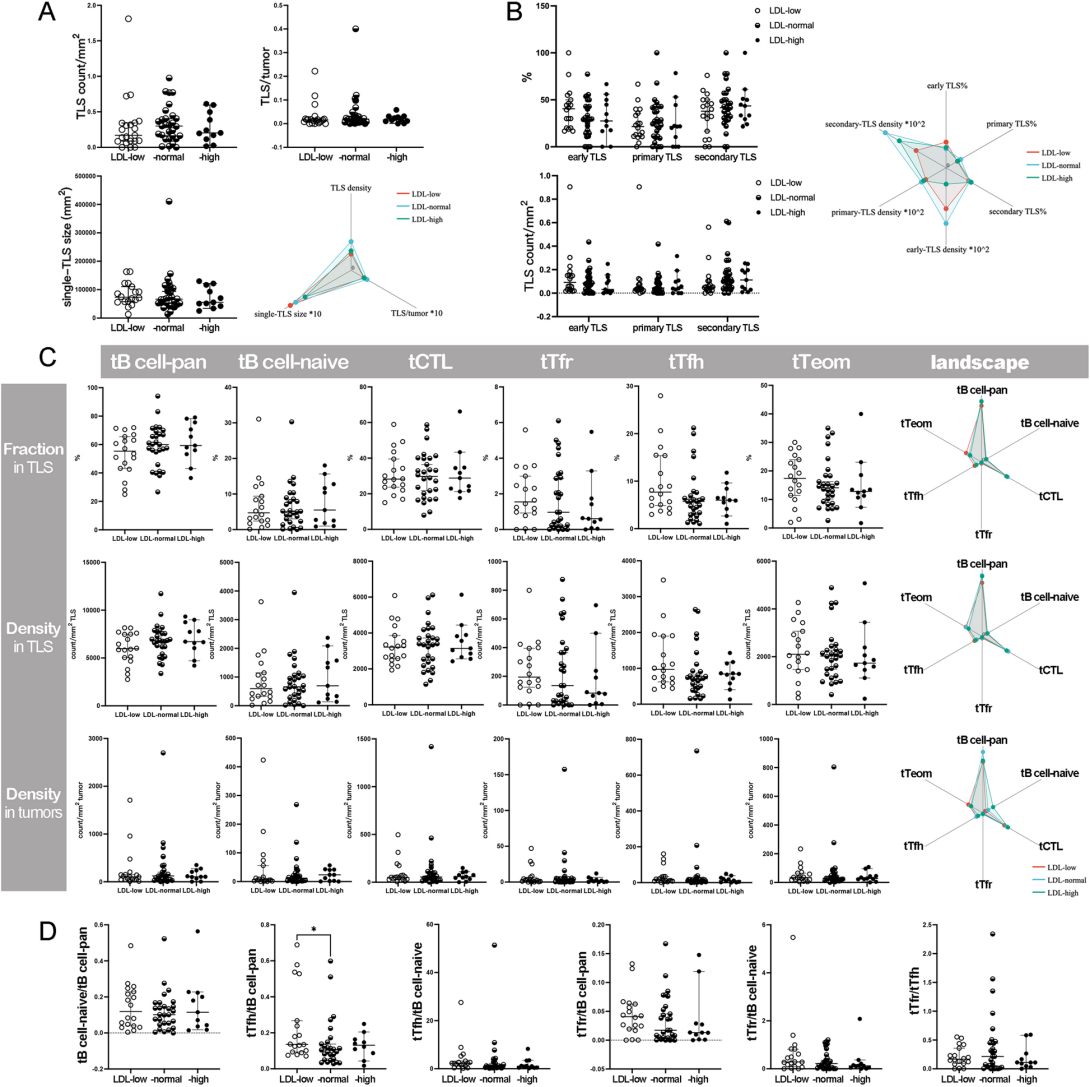
Comparison of TLSs between groups with differential LDL-C levels (**A**) Comparisons of TLS basic features; **B** Comparison of TLS maturation; **C** Comparison of tICs; **D** Comparison of immune ratios. *P < 0.05

The TIME parameters of the LDL-low or -high group were compared with those of the LDL-normal group separately. There was no significant difference between the groups in TLS basic features, including TLS count, TLS density, and single-TLS size (Fig. 4 A), and the maturation of TLSs did not vary with LDL-C levels (Fig. 4B). The comparisons regarding the fractions and densities of tB cell-pan, tB cell-naïve, tCTLs, tTfr cells, tTfh cells, and tTeom cells did not identify any notable difference (Fig. 4 C). However, one immune ratio, tTfh/tB cell-pan, significantly increased in the LDL-low group, while other ratios were comparable between groups (Fig. 4D).

**Fig. 5:**
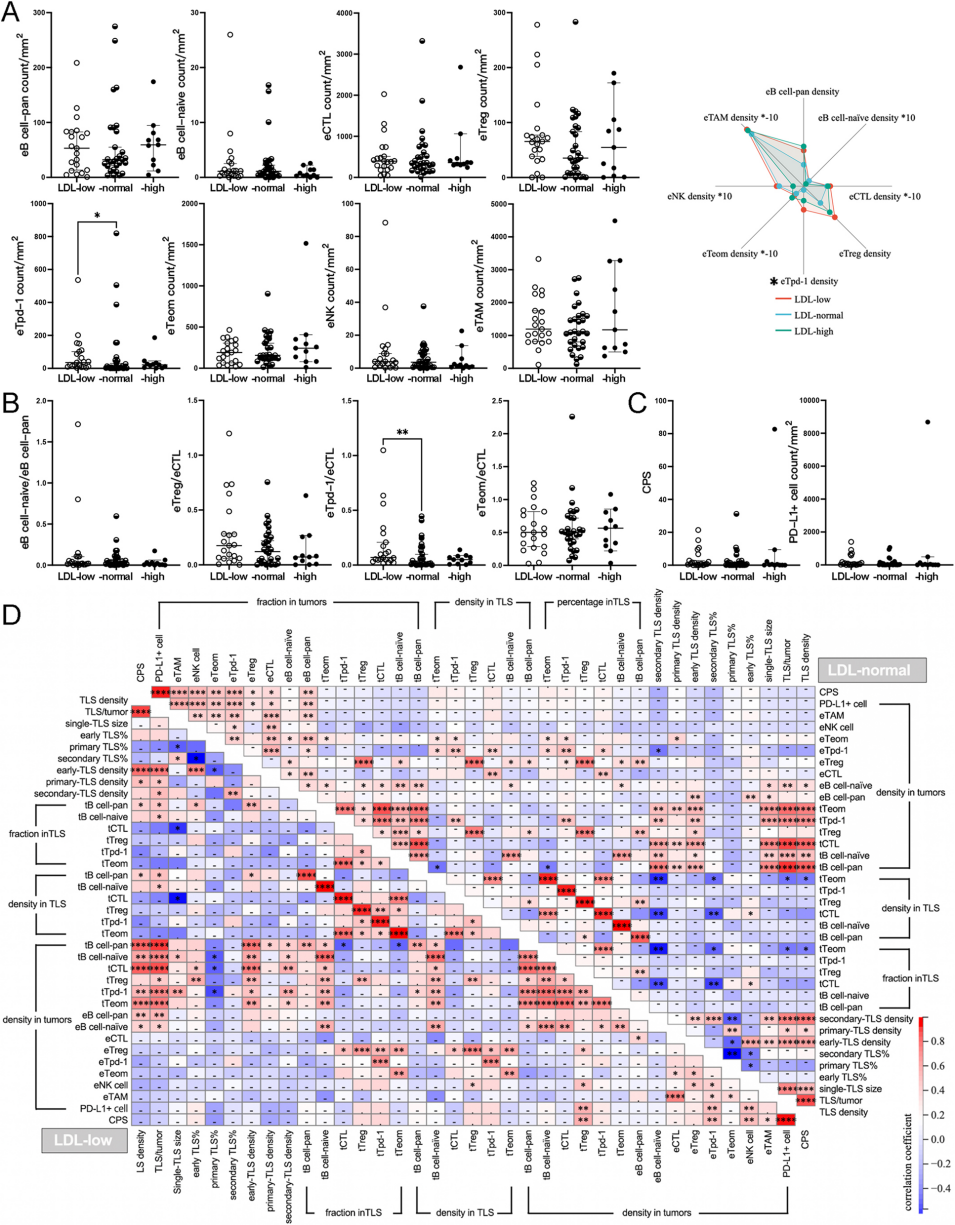
Comparison of the extra-TLS zone between groups with differential LDL-C levels and correlation analysis (**A**) Comparison of eICs; **B** Comparison of immune ratios; **C** Comparison of PD-L1 expression; **D** Correlations between TIME parameters in the LDL-low or -normal group. *P < 0.05, **P < 0.001

In the comparisons of eICs, a remarkable increase in exhausted extra-TLS T cells was found between the LDL-low and LDL-normal groups: eTpd-1 cell density was significantly higher in the LDL-low group (Fig. 5 A), where the ratio of eTpd-1/eCTL was elevated more prominently (Fig. 5B). Otherwise, the densities of other eICs and immune ratios were comparable between the LDL-normal and -low or -high groups. There was no significant difference in PD-L1 expression (Fig. 5 C) or the distribution of immune phenotypes between groups (Supplementary Table 2).

Due to the small sample size of the LDL-high group, the correlation analysis was only performed in the LDL-low and -normal groups. However, similar to the groups with differential HDL-C levels, many common correlations existed in the groups with differential LDL-C levels, within one immune compartment or between two compartments, and correlations exclusive to one group were also seen. For example, in the LDL-low group, tTfr cell density in tumors was correlated with more variables, such as the fraction of early TLSs, the density of eNK cells or PD-L1 + cells, and CPS, while in the LDL-normal group, PD-L1 expression showed broader connections with other variables of the extra-TLS zone (Fig. 5D).

## Weak immune response to tumor progression in patients with low lipoprotein cholesterol

To evaluate the relationships among lipoprotein cholesterol, TIME, and tumor behaviors, we compared the immune parameters between (among) different demographic or clinicopathological variables under differential lipid level scenarios.

Among the significant differences, most were in groups with normal lipid levels, whether in groups divided by HDL or LDL. Differential immune parameters were more frequently found between (among) patients with different tumor invasion, pStage, tumor size, or histological differentiation. The TLS parameters, such as TLS/tumor, tCTL fraction in TLSs, and the density of TLSs or secondary TLSs, changed most prominently along with different demographic and clinicopathological variables, exhibiting a declining trend with tumor progression. The extra-TLS-zone parameters, including the densities of eICs and PD-L1 expression, were significantly affected by tumor location, histological differentiation, and pStage in the HDL- or LDL-normal group, and differing from the TLS parameter trend, peaks of most extra-TLS-zone parameters occurred at the middle stage of tumor progression rather than the early stage (Fig. 6 A and B).

**Figure:**
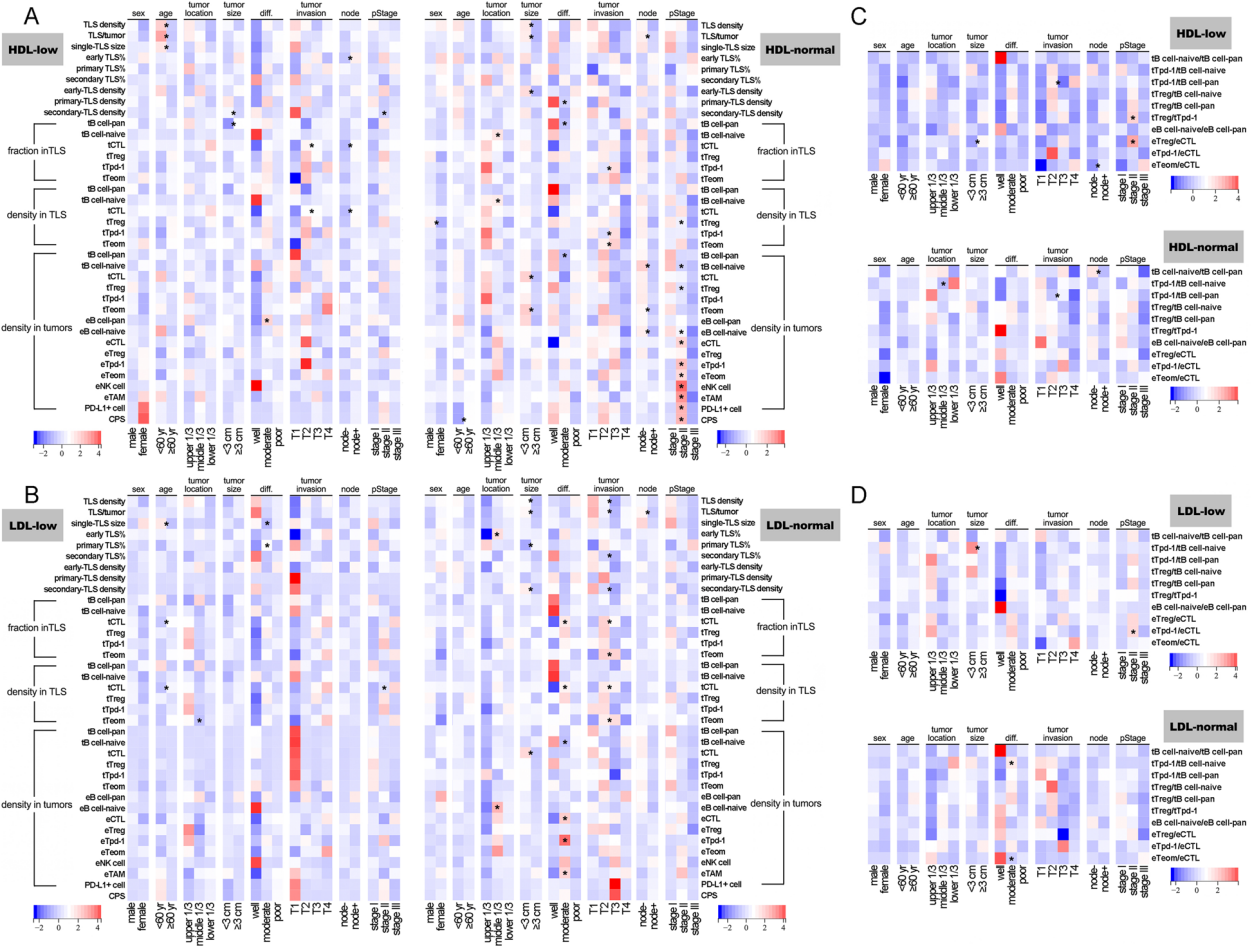
**Fig. 6** Comparison of TIME features among different demographic and clinicopathological features (**A**) Comparison of TIME parameters among different demographic and clinicopathological features in groups with differential HDL-C levels; **B** Comparison of TIME parameters among different demographic and clinicopathological features in groups with differential LDL-C levels; **C** Comparison of immune ratios among different demographic and clinicopathological features in groups with differential HDL-C levels; D Comparison of immune ratios among different demographic and clinicopathological features in groups with differential LDL-C levels. *P < 0.05

Compared to the original values, there were fewer differences in the immune ratios between (among) patients with different demographic or clinicopathological variables under the scenarios with either normal or low lipid levels. However, ratios regarding TLSs also varied more prominently along with tumor progression in a similar trend to the original TLS parameters.

## Discussion

To the best of our knowledge, this is the first study on the relationship between serum lipoprotein cholesterol and the local immune microenvironment in GC with comprehensive approaches covering the two major immune compartments: the TLS and extra-TLS zones. Our study first showed that the immune basis for the antitumor response, including TLS maturation, function, and T-cell exhaustion in the extra-TLS zone, was closely correlated with serum lipoprotein cholesterol. Either low HDL-C or LDL-C levels weaken the dynamically enhanced antitumor immune response along with tumor progression.

As highly specialized and organized local immune structures, TLSs provide sites for B-cell maturation where essential immune cell interactions occur and germinal centers form. The significance of TLS formation has been widely reported. However, increasing evidence has shown that TLS maturation might be more significant for their antitumor function [28]. In the development of TLSs, follicular dendritic cells act as antigen-presenting cells, with CD23 and/or CD21 expressed at the secondary or primary stage [31]. Hence, in the present study, we used CD21 and CD23 staining to determine the stages of TLSs and found that TLS maturation was significantly delayed in GC patients with low HDL-C, which was reported for the first time.

Physiologically, HDL-C is essential for reverse cholesterol transport from peripheral tissues to the liver, and its level has also been found to be significantly correlated with GC occurrence and progression [24–26]. As the major protein component of HDL-C, apoA1 possesses the function of immune modulation. Guo et al. [32] evaluated the density of tumor-infiltrating T cells in colorectal cancer and found that CD3 + T-cell density was significantly higher in patients with elevated serum apoA1 levels, but the distribution of CD8 + T cells was comparable. Zamanian-Daryoush’s study [33] on the murine malignant melanoma model revealed that, compared to apoA1-deficient mice, human apoA1 transgene mice exhibited reduced tumor burden, decreased metastasis, and enhanced survival, which was associated with the increased infiltration of M1 macrophages. More importantly, they demonstrated that apoA1 or HDL only showed their antitumor effect in vivo through innate and adaptive immunity arms, rather than direct suppression. In Wilhelm’s research [34], the number of Treg cells in the lymph nodes of apoA1-/- and LDLr-/- mice was significantly higher than that in the lymph nodes of LDLr-/- mice.

In line with Guo’s work, we did not find a difference in CD8 + T-cell density in the extra-TLS zone, but the distribution of eTAMs, eTregs, and other immune cells was also comparable in our study, which could hardly explain the delayed TLS maturation in patients with low HDL-C levels. However, the strong negative correlation between secondary TLSs and tTfr cells only found in the HDL-low group, and the positive correlation between secondary TLSs and tTfh cells exclusive to the HDL-normal group might give us some hints. Although mainly composed of B cells, the B-cell zone of a TLS also harbors several populations of T cells, such as tTfh and tTfr cells, which play crucially important and counterbalanced roles in TLS maturation [35]. Briefly, as a PD-1 + subset of T cells in lymphoid follicles [36, 37], tTfh cells assist in the proliferation, differentiation, and positive selection of higher-affinity B cells [35]. In contrast, tTfr cells are a FOXP3 + inhibitory subset of T cells in follicles that negatively regulate germinal center formation or antibody production [38] and disturb the activation and function of tTfh cells [39, 40]. Therefore, based on this, the correlations found in our study suggested that, in patients with low HDL-C levels, tTfr cells might predominate the immune balance with tTfh cell inhibition, which restrained B-cell differentiation, leading to delayed TLS maturation. Conversely, the positive correlation between secondary TLSs and tTfh cells in the HDL-normal group supported the balance of preferred tTfh cells, which provided a healthy immune network for TLS maturation.

In our study, NCR1 + eNK-cell density increased unexpectedly in the HDL-low group, which seemed inconsistent with the TIME contexture found here or the patient outcome reported in other studies regarding HDL-C [41]. NCR1 is an activating receptor expressed by pan-NK cells, including resting and activated NK cells [42]. As essential innate immune cells, NK cells contribute significantly to antitumor immunity. However, an inhibitory subset named tolerant NK cells also exists, induced by IL-10 and TGF-beta, and works in an anti-inflammatory pattern [43]. It has been shown that strong correlations between low serum HDL-C and high IL-10 levels exist in various populations [44–47]. Therefore, the contradiction between the higher eNK-cell density and more malignant tumor behaviors might result from the increase in tolerant NK cells in patients with low HDL-C, which were induced by their elevated IL-10 levels. In addition, the positive correlations between eNK cells and eTpd-1 cells, eTreg cells, or PD-L1 expression in the HDL-low group indicated a concurrent increase in exhausted or inhibitory T cells and PD-L1 expression, which also supports the hypothesis regarding IL-10 here, as IL-10 functions widely in T-cell exhaustion and PD-L1 induction [48–51].

The harm of elevated LDL-C levels has been well documented in GC occurrence. It showed that higher LDL-C levels significantly increased the risk of GC [24], and phytosterols or HMG CoA reductase inhibitors could help inhibit GC development by reducing serum LDL-C levels [6, 8]. However, in patients who already have GC, due to decreased food intake or excessive energy consumption by tumors, hypolipidemia or other malnutrition is common [52], even at the early stage [53]. This was consistent with our cohort, in which low LDL-C levels were observed in more than 1/3 of patients. Hence, the TIME features of the LDL-low group received more attention here, and data showed that low LDL-C levels indeed correlated with unfavorable immune changes: the ratio of tTfh/tB cell-pan or eTpd-1/eCTL and eTpd-1 cell density increased significantly. Based on the process of TLS maturation mentioned above, these findings suggested that in patients with low LDL-C, the ability of tTfh cells to help B-cell maturation was impaired, and more T cells were exhausted in the extra-TLS zone.

It was demonstrated that T-cell activities depend highly on the amount of membrane cholesterol to cluster T-cell receptors (TCRs) and form immune synapses [20]. In tumors, a hostile metabolic microenvironment is created, making T cells lose their ability to synthesize lipids [19], and the exogenous lipid supply becomes critical. Muldoon et al. [54] showed that hypolipidemia (LDL-C ≤ 110 mg/dL) significantly lowers the count of circulating lymphocytes, total T cells, or CD8 + T cells. In Waelti’s research [55], elevated LDL-C markedly enhanced T-cell activation in vitro. Furthermore, Yuan et al. [56] found that obtaining cholesterol from LDL-C via LDL receptors was indispensable for the activation and clonal expansion of CD8 + T cells. The antitumor effect of CD8 + T cells was significantly improved by the interaction between LDL receptors and TCRs. However, the relationship between LDL-C and T-cell exhaustion is ill defined. Ma’s work [57] reported that excessive cholesterol in the tumor microenvironment promoted CD8 + T-cell exhaustion by increasing endoplasmic reticulum stress. Our study showed that low LDL-C levels were also associated with increased T-cell exhaustion in GC. The lack of cholesterol supply might be a key factor here, which is also a probable reason for the dysfunction of tTfh cells.

In addition, along with tumor progression, we also noticed more remarkable cell dynamics in TLSs and earlier peaks of TLS-related parameters than in the extra-TLS zone. This phenomenon might indicate that TLSs were not only the “local schools” for immune cells but also the “sentinel points” for immune surveillance and the “spearheads” for quick responses to tumors. This is consistent with Yamakoshi’s finding [15] that TLSs participate in the initiation of T-cell and B-cell responses in GC. Moreover, the weaker immune dynamics in patients with low lipoprotein cholesterol emphasize the imperative role of lipids in antitumor immunity again and strengthen the position of lipid management in GC patient.

## Study strengths and limitations

This study disclosed the TIME differences among GC patients with differential serum cholesterol levels based on whole-tissue-section analyses, and indicated the potential significance of serum lipids in antitumor immune response. However, there were several limitations of this study. First, although IHC staining is an economical and convenient approach, the coexpression of some markers might affect cell type identification to some extent. Second, although serial sections were adopted, cell count and arrangement might vary slightly among different sections, affecting cell fraction or density comparisons. Third, due to the heavy work to annotate every TLS in all tumor regions, the sample size was relatively small here, and subgroup analyses and the interaction assessment of HDL- and LDL-C were restricted. Hence, in the future, multiplex IHC staining should be applied in large-scale cohorts to further evaluate the TIME, and artificial intelligence approaches are needed to make TLS annotation and measurement more efficient.

## Conclusion

In summary, the present study first reported the relationship between serum lipoprotein cholesterol and TIME features of GC from the aspects of two immune compartments. Our data showed that low lipoprotein cholesterol significantly affected antitumor immunity in TLSs or the extra-TLS zone. Normal lipoprotein cholesterol levels are imperative for cell dynamics in the immune response to tumor progression. These findings emphasize the importance of lipid or nutrition management in GC patients and provide preliminary directions for developing drugs targeting immunometabolism.

## Supplementary Information

**Additional file 1.**

## Data Availability

The datasets used and analyzed during the current study are available from the corresponding author on reasonable request.

## Abbreviations

GC: gastric cancer
HDL-C: high-density lipoprotein cholesterol
LDL-C: low-density lipoprotein cholesterol
PSM: propensity-score matching
TIME: tumor immune microenvironment
IHC: immunohistochemistry
TLS: tertiary lymphoid structure
ROI: regions of interest
tIC: intra-TLS immune cell
eIC: extra-TLS immune cell
tTfh: T follicular helper cell
tTfr: T follicular regulatory cell
CTL: cytotoxic T lymphocyte
Treg: T regulatory cell
TAM: tumor-associated macrophage
NK cell: natural killer cell
Tpd-1: PD-1+ T cell
Teom: EOMES+ T cell
TCR: T-cell receptor
PD-L1: programmed death-ligand 1
CPS: combined positive score
