# Supplementary material for: Adverse effects of low serum lipoprotein cholesterol on the immune microenvironment in gastric cancer: a case‒control study
Source: Lipids Health Dis. 2022 Dec 31;21:150. doi: 10.1186/s12944-022-01766-z (PMC9805280; doi:10.1186/s12944-022-01766-z)
Supplement: Supplementary file 1 — Additional file 1. [file 12944_2022_1766_MOESM1_ESM.docx]

Supplementary Table 1. Comparison of TLS distribution between HDL or LDL groups

|  |  | TLS | |  | Location | | |
| --- | --- | --- | --- | --- | --- | --- | --- |
|  |  | Absence | Presence |  | Intratumoral | Stromal | Peritumoral |
| HDL-C | Low | 3 (13.0) | 20 (87.0) |  | 2 (10.0) | 8 (40.0) | 10 (50.0) |
|  | Normal | 1 (4.3) | 22 (95.7) |  | 2 (9.1) | 11 (50.0) | 9 (40.9) |
| P | | 0.608 | |  | 0.900 | | |
| LDL-C | Low | 3 (14.3) | 18 (85.7) |  | 3 (16.7) | 7 (38.9) | 8 (44.4) |
|  | Normal | 1 (3.4) | 28 (96.6) |  | 2 (7.1) | 12 (42.9) | 14 (50.0) |
|  | High | 0 | 11 (100.0) |  | 1 (9.1) | 7 (63.6) | 3 (27.3) |
| P^†^ | | 0.297/1.000 | |  | 0.639/0.412 | | |

† LDL-low vs LDL-normal / LDL-high vs LDL-normal

Supplementary Table 2. Comparison of immune phenotype distribution

|  | Immune dessert | Immune-excluded | Inflamed | P |
| --- | --- | --- | --- | --- |
| HDL-C |  |  |  |  |
| Low | 4 (28.6) | 6 (42.9) | 4 (28.6) | 1.000 |
| Normal | 5 (33.3) | 5 (33.3) | 5 (33.3) |  |
| LDL-C |  |  |  |  |
| Low | 2 (13.3) | 9 (60.0) | 4 (26.7) | 0.074/0.797^†^ |
| Normal | 9 (45.0) | 5 (25.0) | 6 (30.0) |  |
| High | 1 (25.0) | 1 (25.0) | 2 (50.0) |  |

† LDL-low vs LDL-normal / LDL-high vs LDL-normal
